# Evaluation of cost-effectiveness of single-credit traffic safety course based on Kirkpatrick model: a case study of Iran

**DOI:** 10.1186/s12909-024-05122-w

**Published:** 2024-02-09

**Authors:** Mina Golestani, Homayoun Sadeghi-bazargani, Sepideh Harzand-Jadidi, Hamid Soori

**Affiliations:** 1 Road Traffic Injury Research Centre, Tabriz University of Medical Sciences, Tabriz, Iran; 2 Department of Epidemiology and Biostatistics, School of Health, Tabriz University of Medical Sciences, Tabriz, Iran; 3 Safety Promotion and Injury Prevention Research Center, Department of Epidemiology, School of Public Health and Safety, Shahid Beheshti University of Medical Sciences, Tehhran, Iran

**Keywords:** Cost-effectiveness, Evaluation, Kirkpatrick, Road traffic accidents, Traffic safety

## Abstract

**Background:**

Training plays a role in reducing traffic accidents, and evaluating the effectiveness of training programs in managers’ decision-making for training continuation is important. Thus, the present study aimed to evaluate the cost-effectiveness of a single-credit traffic safety course based on the four levels of the Kirkpatrick model in all Iranian universities.

**Methods:**

This interventional study aimed to evaluate the cost-effectiveness of a single-credit traffic safety course based on the Kirkpatrick model from 2016 to 2020 in Iran. The data were collected in three stages: (1) calculating the costs of offering traffic safety courses, (2) determining the effectiveness of providing such courses based on the levels of the Kirkpatrick model, and (3) evaluating the cost-effectiveness of administering traffic safety courses. Data were collected through researcher-made and standardized questionnaires. The research population included traffic safety course instructors and university students who could take this course. Finally, the data were analyzed with SPSS v. 23 and also calculations related to ICER, which shows the cost effectiveness of providing single credit course.

**Results:**

Scores of the students’ reaction level to the traffic safety course was 41.8% before the course; this score was estimated at 67% after the course. At the level of learning, students’ knowledge was 43.6% before the training course, which reached 73% after the course. At the level of behavior, the state of students’ desirable traffic behaviors was 54% before the course, which reached 66.1% after the course. The educational effectiveness of the course presentation at the level of results was 58.2% before and 74.8% after the course. While assuming that the weights of all model levels were constant, the cost of a 1% increase in the overall educational effectiveness by using the Kirkpatrick model, compared to not providing the course (not administering the intervention) was 486.46 USD.

**Conclusion:**

The results showcased the effectiveness of the traffic safety course in all four levels of The Kirkpatrick model. Therefore, policy-makers and officials in charge of delivering this program should strengthen it and resolve its deficiencies to realize all its educational goals at the highest level.

## Introduction

Traffic accidents are the leading cause of injuries and the second cause of death in Iran. The examination of human factors involved in accidents in Iran shows that most of these factors can be resolved to some extent with training. Sustainable traffic safety training in the Netherlands, Spain, and Germany partly explains the success of these countries in improving traffic behavior and reducing traffic accidents [1, 2]. Therefore, the role of training in reducing traffic accidents is undeniable, but traffic safety training is not beneficial on its own [2, 3]. A training program can justify its value when it provides reliable and valid evidence about the effects of training on improving the learners’ behavior and performance. As such, it is necessary to evaluate educational programs [4, 5].

Educational program evaluation informs educational planners and staff about the quality of education. It also helps them become aware of the positive and negative aspects of the educational program and, thus, make educational programs and activities more effective [6]. Various approaches have been presented to evaluate the effectiveness of educational programs, including the Phillips, Kirkpatrick, and Sullivan [7]. The Kirkpatrick model is one of the most comprehensive and practical evaluation models for educational programs, which defines evaluation as determining the effectiveness of an educational program [8].

In this model, four levels are proposed for educational evaluation: (1) reaction level (measures how learners feel about all the factors affecting the delivery of the educational program), (2) learning level (determining the level of acquisition of skills, techniques, and facts taught and explained to the participants in the course, and can be understood through training before, during, and after participating in the course), (3) behavior level (the type and degree of changes in the behavior of participants as a result of participating in training courses), and (4) results level (the level of achievement of goals directly related to the organization) [9, 10]. Numerous studies have evaluated educational programs with the Kirkpatrick model. Campbell et al. examined the effect of online cancer training on the level of knowledge and efficiency of nurses based on the Kirkpatrick model. Lillo-Crespo et al. used this model to study the effect of advanced healthcare training on the level of knowledge of students. Akbari et al. explored the impact of holding an in-service cardiopulmonary resuscitation training course on the knowledge level of 80 nurses based on the said model [11–13]. Therefore, this model can be adopted to evaluate educational courses in different fields.

In various organizations, vast sums of money are spent on training employees’ specific skills, while in most cases, the effectiveness of training is not measured, and proper feedback from learners is not obtained. By evaluating the courses, it is possible to judge to what extent the performance of educational programs has been desirable and to what extent it should be improved. It can also be judged how effective this program was and whether the expenses spent on the training were economically justified. Unfortunately, in the Iranian educational system, in many cases, the effectiveness evaluation system either does not exist or is disorderly or rudimentary [6, 14]. Considering the importance of evaluating the effectiveness of educational programs in managers’ decision-making for the continuation of training, the present study aimed to evaluate the cost-effectiveness of a single-credit traffic safety course based on the four levels of The Kirkpatrick model. Using the results of the evaluation presented by this study, it is possible to apply the necessary reforms in the planning and delivery of this course and propose practical solutions to improve the quality of its delivery. If the desired outcomes are achieved, the final syllabus of the single-credit traffic safety course will be developed and presented to the Supreme Council of Cultural Revolution to be taught as a suitable and suggested model in all Iranian universities.

## Materials and methods

To promote the RTAs-related knowledge in Iran, the task of developing traffic knowledge was assigned to the 2nd Territorial Agenda to carry out the relevant arrangements according to the needs announced by the Ministry of Health, Treatment and Medical Education and considering the potential of research fields and suitable background in this field, the specialized authority for the development of traffic knowledge and road accidents in the 2nd region of Establishment of Territorial Agenda after signing a joint memorandum with the Ministry of health, officially started his activity with the center of Tabriz University of Medical Sciences. Therefore, single-credit course of traffic safety was decided to present for the first time as a compulsory pilot course in volunteering universities. For this purpose, all experts in the field of traffic knowledge were invited and participated, then the educational curriculum of the course was designed, after the approval of this curriculum in the Ministry of Health and Medical Education, this course was presented for 5 years in the universities of medical sciences in the country.

This course was implemented for all students of all academic levels. Eight sessions were held during one academic semester. Each session lasted two hours. Of course, the classes were held both theoretically and practically, especially for the first aid part. The training was done in a hybrid way. For trainers due to the scope of the program, it is difficult to use traffic experts to teach this subject. In this regard four training of trainers (TOT) programs were performed. For students, three-fourths of the classes were completely face-to-face and one-fourth of the classes were held online.

This interventional study aimed to determine the cost-effectiveness of a single-credit traffic safety course in Iran in five years (from 2016 to 2020). The research population consisted of two groups: (1) a total of 2066 students of 12 Iranian universities of medical sciences across the country and (2) professors of these universities. The inclusion criteria for students were passing a single-credit traffic safety course and willingness to participate. The inclusion criteria for the professors were experience teaching this course and willingness to participate. Students and professors who did not wish to continue participating in the study for any reason or whose information was incompletely recorded were excluded.

To attain the primary goal of the study (cost-effectiveness evaluation), first, the costs of each component of the educational program were determined, and then, the cost-effectiveness of this course was evaluated by determining the effectiveness of each level of The Kirkpatrick model:

### Calculating the total costs of offering a single-credit traffic safety course in Iran

To analyze the costs of holding the course compared to its effectiveness, first, all the costs spent on the delivery of the traffic safety training program (including the costs of developing and improving human resources, the costs of providing infrastructure, the costs related to the preparation, production, and presentation of educational materials, the costs of teaching and learning process) were calculated the by step down method.

### Investigating the educational effectiveness of the traffic safety course based on four levels of The Kirkpatrick model

The Kirkpatrick model was employed to evaluate the effectiveness of the course; in fact, it measured the degree of realization of the educational goals of the course and the effectiveness of the measures taken to achieve its goals. For this purpose, the effectiveness of the traffic safety course was explored at four levels once before the implementation of the course and then 6 months after the implementation of the course was re-evaluated.

The students were surveyed before and after the traffic safety course to check the reaction level. In this survey, 2066 students from 12 universities participated and filled out a researcher-made questionnaire with 9 questions. This instrument examined the participants’ feelings about participating in the course and their degree of satisfaction with the course content. The validity of the questionnaire was confirmed, with a content validity index (CVI) of 0.84, and its reliability was confirmed with an intra-class correlation coefficient (ICC) of 0.81.

The test-retest method was used to check the learning level. To this end, the traffic knowledge level of the students was assessed in the first session of the course using a researcher-made questionnaire. The validity of the questionnaire was confirmed with the content validity index (CVI) and content validity ratio (CVR), which were above 80%, and its reliability was confirmed with the correlation coefficient (ICC).

Then, after 6 months, in the last session, this test was re-administered using the same questionnaire to the same students. A sample of 1056 students was considered for assessing the impact of the course; of these, some students were evaluated only once and had incomplete information. Therefore, 293 students were excluded, and finally, the data of 763 students were analyzed. The instrument designed to evaluate the impact of the training course included 19 questions about different areas of traffic. Out of 19 questions, two descriptive questions dealt with the symptoms of sleep apnea and key points about environmentally friendly driving; 17 tests examined the students’ traffic knowledge in 5 general domains: safety improvement and epidemiology (4 items), pedestrian safety (2 items), first aid (2 items), vehicle safety standards (3 items), road and special traffic issues (6 items).

In order to check the level of behavior (i.e., the manner and degree of changes in traffic behavior) before and six months after the course, the Pedestrian Traffic Behavior Questionnaire designed by Haghighi et al. [15] was administered to the 515 students under study. This questionnaire included 29 items that measured pedestrian behaviors in five dimensions (following rules, violations, positive behaviors, distractions, and aggressive behaviors) on a 5-point Likert scale. Higher scores indicated safer behavior.

In examining the level of results, the views of 25 instructors about the impacts of the traffic safety course and the results of its presentation were examined in interviews before and after the course (6 months after the implementation of the program). The instrument used for these interviews was a researcher-made questionnaire that examined the level of achievement of the goals of the Ministry of Health and Medical Education and the expert authority on traffic knowledge development.

After collecting data at each level, the data were inputted to SPSS v. 23. Data analysis in the effectiveness section was performed with descriptive statistics (mean, standard deviation, frequency, and percentage) and statistical tests, such as paired t tests.

### Evaluating the educational cost-effectiveness of the traffic safety course based on the Kirkpatrick model

The Cost-effectiveness analysis (CEA) technique for educational interventions was used to evaluate the cost-effectiveness of the traffic safety course. CEA measures the relationship between a project’s total inputs or costs and its outputs or objective results. In this technique, both cost and effectiveness dimensions should be quantitatively determined. CEA can be performed in two ways: (a) comparing different strategies and ways to reach a goal and determining the best strategy by simultaneously considering the costs and outcomes of each strategy; this method can be applied in education through a comparison between educational centers, types of education, or different teaching methods. (b) comparing two or more faculties, universities, and even instructors who behave with almost the same costs but different educational effectiveness and determining the most effective educational institution or instructor. In both cases, we must determine, measure, and calculate the incremental cost-effectiveness ratio (ICER) for each strategy or educational institution/instructor. This ratio determines the additional cost of achieving a higher output by a strategy compared to other strategies.

The following equations were used for the CEA: CER = C/E.

Average cost-effectiveness = the costs of offering the course divided by the model’s effectiveness levels / Educational effectiveness divided by the model’s effectiveness level

ICER = (costs before administering the educational course – costs after administering the educational course) / Educational effectiveness before the course – educational effectiveness after the course.

## Results

### Overall costs of presenting the single-credit traffic safety course at the national level

Based on Table 1, the greatest share of the costs of course presentation belonged to the development and improvement of human resources, the teaching-learning process, and the development of teaching materials, respectively.

**Table 1:** Costs of offering the traffic safety course

Cost items	Total cost (USD)	Cost share percentage
Costs of developing and improving human resources	16,704.68	37
Costs of providing the infrastructure	2,963.92	7
Costs of developing, producing, and presenting the educational materials	12,804.15	28
Costs of the teaching-learning process	13,060.24	29
Total	45,533.01	100

### Educational effectiveness of the traffic safety course based on four levels of The Kirkpatrick model

#### Reaction level

Out of 2066 students participating in this section, most students belonged to Sabzevar (36.93%) and Tabriz (32.28%) universities of medical sciences. Table 2 shows the results of the students’ survey about the traffic safety course for each item. The results show that most students had moderate and moderate-to-high satisfaction in all items. Based on the reaction level results, the score of the students’ reaction level to the traffic safety course was 41.8% before the course; after the course, this score was estimated at 67%.

**Table 2:** Students’ satisfaction with the delivery of the traffic safety course (after the course)

Item	Student’s satisfaction with the traffic safety course: N (%)				
	Very low	Low	Moderate	high	Very high
Necessity of including the course in the curriculum	286(13.88)	199(9.66)	544(26.41)	578(28.06)	453(21.99)
Necessity of learning the educational content	163(7.90)	168(8.15)	520(25.22)	724(35.11)	487(23.62)
Motivation for further learning about traffic	254(12.35)	299(14.54)	593(28.84)	539(26.22)	371(18.04)
Necessity of offering the course as a general course	116(5.64)	163(7.93)	489(23.78)	754(36.67)	534(25.97)
Application of the course in personal life	121(5.88)	151(7.34)	499(24.26)	780(37.92)	506(24.60)
Effect of the course on social life (promoting safe behaviors in people’s lives)	133(6.48)	135(6.58)	547(26.64)	772(37.60)	466(22.70)
Effect of course delivery on traffic behavior	155(7.56)	161(7.85)	549(26.78)	729(35.56)	456(22.24)
Effect in shaping expectations of authorities in demanding civil rights	205(9.99)	186(9.06)	572(27.86)	629(30.64)	461(22.45)
Total satisfaction with the course presentation	290(14.29)	226(11.13)	554(27.29)	561(27.64)	399(19.66)

#### Learning level

A total of 763 students from 25 fields of medical sciences participated in this part, most of whom belonged to allied health sciences (37.35%). About half of the participants were undergraduate students (56.23%), and most participants were from Sabzevar (39.29%) and Tabriz (26.08%) universities of medical sciences. According to Table 3, which presents the students’ traffic knowledge status before and after the course, the students’ total knowledge and their traffic knowledge in different dimensions increased strongly and significantly after the course (*p* < 0.001). Based on the results of the learning level of The Kirkpatrick model, the students’ knowledge level was 43.6% before the course; after the course, it was estimated at 73%.

**Table 3:** Students’ traffic knowledge scores before and after the training course (*n* = 763)

Domain	Measurement time	Mean and SD	Median (min-max)	*p*-value
Total	Before	43.64(13.88)	41.18(0-82.35)	< 0.001
	After	69.61(17.28)	70.59(5.88–100)	
Introduction to safety improvement and epidemiology	Before	44.33(23.37)	50(0-100)	< 0.001
	After	69.61(18.42)	75(0-100)	
Pedestrian safety	Before	48.62(28.55)	50(0-100)	< 0.001
	After	71.17(30.75)	50(0-100)	
First aid	Before	56.49(32.00)	50(0-100)	< 0.001
	After	80.08(28.11)	100(0-100)	
Vehicle and road safety standards	Before	28.63(23.94)	33.33(0-100)	< 0.001
	After	64.14(32.55)	66.67(0-100)	
Specific traffic issues	Before	44.69(20.29)	50(0-100)	< 0.001
	After	71.97(23.13)	66.67(0-100)	

#### Behavior level

Based on the results of examining the educational effectiveness at the behavior level, the state of students’ desirable traffic behaviors before the course was 54%, which reached 66.1% after the course. This behavior change was 64% in following the rules, 58% in violations, 61% in positive behavior, 65% in distraction, and 90% in violent behaviors.

#### Results level

Based on the interviews with traffic safety course instructors, most of the instructors (75%) believed that offering this course greatly impacted the students’ social behavior and would continue to exert its effects in the future, too. Most of the instructors (84%) believed that the course greatly influenced the students’ personal lives. About 80% of the instructors believed that the course’s content greatly helped improve the students’ safety. About 71% of them held that offering the course to students could effectively shape their expectations of officials in demanding civil rights. Besides, 64% of instructors said this course motivated students to learn more about traffic. In general, the educational effectiveness of course delivery at the level of results was 58.2% before the course, which reached 74.8% after the course.

### The cost-effectiveness of offering a traffic safety course based on the Kirkpatrick model

Table 4 shows the average educational cost-effectiveness ratio (CER) based on Kirkpatrick model levels. According to the results, the average cost per 1% educational effectiveness is 293.56 USD at the reaction level, 354.31 USD at the learning level, 436.79 USD at the behavior level, and 61.52 USD at the results level.

**Table 4:** The average educational cost-effectiveness of offering the traffic safety course based on the levels of the Kirkpatrick model

Levels of the Kirkpatrick model	Educational effectives after the course (%)	Cost items	Estimate cost	Total cost by levels	Cost-effectiveness ratio (USD)
Reaction level	67	Costs of developing and improving human resources	16,704.68	19,668.61	293.56
		Costs of providing the infrastructure	2,963.92		
Learning level	73	Costs of developing, producing, and presenting the educational materials	12,804.15	25,864.4	354.31
		Costs of the teaching-learning process	13,060.24		
Behavior level	66	Total Cost of learning level		28,828.32	436.79
		Costs of providing the infrastructure			
Results level	74.8	Total Cost of reaction level		45,533.01	61.52
		Total Cost of learning level			

Table 5 presents the educational incremental cost-effectiveness ratio (ICER) of offering the traffic safety course based on The Kirkpatrick model. Based on the results, the cost per 1% increase in educational effectiveness is 662.89 USD at the reaction level, 576.02 USD at the learning level, 1,256.31 USD at the behavior level, and 2,742.95 *USD* at the results level. The cost per 1% increase in the overall educational effectiveness obtained by using the Kirkpatrick model (compared to not offering the course (no intervention)) is 486.46 USD (assuming that the weights of all model levels remain constant).

**Table 5:** Incremental educational cost-effectiveness of offering the traffic safety course based on levels of The Kirkpatrick model

Levels of the Kirkpatrick model	Educational effectives (%)		Total cost by levels		Incremental cost-effectiveness ratio (USD)
	Before the course	After the course	Before the course	After the course	
Reaction level	41.8	67	2,963.92	19,688.61	662.89
Learning level	43.6	73	25,864.4	42,569.07	576.02
Behavior level	54	66	13,752.6	28,828.32	1,256.31
Results level	58.2	74.8	0	45,533.01	2,742.95
**Total incremental cost-effectiveness**		**70.2**		**34,149.75**	**486.46**

## Discussion

This study determined the cost-effectiveness of a traffic safety course based on the Kirkpatrick model. The results revealed that offering this single-credit course to students is a cost-effective educational program. The costs imposed on society due to accidents and the resulting injuries are about 7% of GDP (gross domestic product), and the (disability adjusted life years) DALY due to traffic accidents was estimated at about 1738 years of life with premature death and disability per 100,000 people in Iran in 2016. Based on calculating the cost of increasing the effectiveness of this course, the cost per 1% increase in the overall educational effectiveness obtained using The Kirkpatrick model, compared to the non-presentation of the course (no intervention), is 486.46 USD (assuming the constant weights of all levels of the model). In fact, this cost is not high compared to the effectiveness achieved at the levels of the Kirkpatrick model, especially at the level of results. Therefore, the estimated cost due to the delivery of the traffic safety training course, which is about 4,742.28 USD from the share of health system costs to improve its effectiveness by 1%, is not significant compared to the high costs of traffic accidents and injuries. Although providing such programs entails costs, it is an investment in human resources. Especially in promoting a safety culture and developing traffic knowledge, such courses can reduce the high costs of traffic accidents and injuries and lead to considerable economic savings. They also increase the productivity of the labor force, who lose their lives or are disabled daily due to traffic accidents.

The study by Bazarafkan et al. to evaluate the effectiveness of training courses for health volunteers according to the Kirkpatrick model, showed that the training program for health volunteers is effective [16]. Myall’s study also confirmed the effectiveness of the internship mentorship program for nursing students [17].

### Reaction level

At the reaction level, the cost per 1% increase in educational effectiveness was 662.89 USD. At this level, most participants had moderate-to-high satisfaction with the presentation of the traffic safety course and found the course content practical and useful. The educational effectiveness at this level was 41.8% before the course and 67% after the course. This significant increase in effectiveness at the reaction level can be due to the impact of the course content on the students’ personal and social life and behaviors. The results of this level align with the findings of previous studies based on The Kirkpatrick model. Nezamianpour et al. evaluated the reaction of nurses toward the training course on working with electroshock equipment to be favorable [18]. In Mohan’s study, most participants were highly satisfied with the course at the reaction level [19]. In Yoon et al.‘s study, which explored the training program for the professional development of physicians, the learners were satisfied with the course [20]. Akbari et al. also evaluated the reaction of nurses and paramedics to the cardiopulmonary resuscitation course [11].

### Learning level

Based on the study’s results, the cost per 1% increase in educational effectiveness was 576.02 USD at the learning level. At this level, students’ knowledge before the traffic safety course was 43.6%, which was estimated at 73% after the course. According to the results of the present study, passing the traffic safety course significantly increased students’ traffic knowledge in different traffic domains. Students showed the most significant increase in knowledge in vehicle and road safety standards. The significant increase in students’ traffic knowledge can be because the teaching of various traffic safety topics was neglected in the Iranian education system, and the current level of knowledge about such topics is low among people, including students. Therefore, providing practical training can play a key role in improving students’ traffic knowledge. Students can improve society’s knowledge about traffic safety by transferring their traffic knowledge to their family and friends. The results of this level were aligned with the results of Bazarafkan et al. In the cited study, there was a significant difference between the participants’ average scores on the post-test compared to the pre-test, indicating that their level of awareness, knowledge, and skills increased after the health volunteer program [16]. Heidari et al. also measured the impact of a training workshop on new teaching methods for healthcare workers; they showed a significant difference between the learning scores of the participants before and after the course, demonstrating an increase in their learning and knowledge after the course [21]. Hojjati et al. assessed the effectiveness of in-service training courses for nurses based on the Kirkpatrick model, and their results were in line with the results of the present study regarding the learning level [14]. Le et al. evaluated the training program for physicians in Vietnam with the Kirkpatrick model and reported a positive improvement in the participants’ learning and skills [22].

### Behavior level

At the behavior level, the cost per 1% increase in educational effectiveness was 1,256.31 USD. Based on the results of educational effectiveness at this level, the state of desirable traffic behaviors among students was 54% before the course, which reached 66.1% after the course. This change was observed in different aspects of traffic behavior, especially in violent behaviors. Of course, compared to other levels of The Kirkpatrick model, the change in behavior after the course was less (12%). It seems that continuing to provide traffic safety courses, increasing the number of units and hours, and resolving its deficiencies can have a greater impact on changing the behavior of learners. Mollakazemi et al. showed that participating in occupational medicine retraining courses can positively affect changing the behavior of general practitioners [4]. Nega et al.‘s study about the educational program at a faculty of medicine and pharmacy evaluated the third level of The Kirkpatrick model to be positive [22]. In the study by Ranjdoust et al., the behavioral attitudes of trained students were increased in terms of being influenced by what they learned compared to untrained students [23].

### Results level

At the level of results, for a 1% increase in educational effectiveness, the cost was 2,742.95 USD. At this level, the effectiveness was 58.2% before and 74.8% after the course, which is a significant change. The instructors believed that the traffic safety course had a positive effect on various aspects of the students’ lives, including their personal and social lives and their traffic behaviors, and the continuation of this course could exert positive impacts in the future as well. The results of this level of study were in line with the study by Jamaledini et al., who showed that the results of crisis management training courses were effective [24]. In Shayan et al.‘s study, the reduction of pneumonia infection in the ICU of Taleghani Hospital, Tehran, demonstrated the effectiveness of staff training programs [25]. In the study by Dehghani et al., the results of the level of nurses’ behavior and the level of results also showed that most of the training course objectives were attained [2, 26].

### Strengths and limitations

The present study is the first in Iran to evaluate the cost-effectiveness of a single-credit traffic safety course based on the Kirkpatrick model. Administrative bureaucracies were a limitation that affected the program’s effective administration. By attracting the support of relevant officials in this field and holding numerous meetings, we tried to solve the problems related to the administrative bureaucracy. The other limitation was the impossibility of accessing all the students who had passed this course. Additionally, self-reported approach to evaluate the behavior of pedestrians at the behavior level was another limitation in the current study.

## Conclusion

The current study’s findings revealed the effectiveness of the traffic safety course in all 4 levels of the Kirkpatrick model. The majority of learners were satisfied with the presentation of the course. The traffic safety course improved the participants’ traffic knowledge and behavior, and the course content was useful in their personal and social lives. Therefore, policy-makers and authorities in charge of the delivery of this program should improve this course and resolve its deficiencies so that all its educational goals can be realized at their highest level.

## Data Availability

The datasets used and/or analysed during the current study available from the corresponding author on reasonable request.
